# Proctitis following stereotactic body radiation therapy for prostate cancer

**DOI:** 10.1186/s13014-014-0277-4

**Published:** 2014-12-12

**Authors:** Daniel Y Joh, Leonard N Chen, Gerald Porter, Aditi Bhagat, Sumit Sood, Joy S Kim, Rudy Moures, Thomas Yung, Siyuan Lei, Brian T Collins, Andrew W Ju, Simeng Suy, John Carroll, John H Lynch, Anatoly Dritschilo, Sean P Collins

**Affiliations:** Department of Radiation Medicine, Georgetown University Medical Center, 3800 Reservoir Road, N.W, Washington, DC, 20007 USA; Department of Urology, Georgetown University Hospital, Washington, DC, 20007 USA; Department of Gastroenterology, Georgetown University Hospital, Washington, DC, 20007 USA; Department of Radiation Oncology, East Carolina University, Greenville, NC 27834 USA

**Keywords:** Prostate cancer, SBRT, Rectal endoscopy, Telangiectasias, CyberKnife, Expanded prostate index composite, Bother, Proctitis, Rectal bleeding, Vienna rectoscopy score

## Abstract

**Background:**

Proctitis after radiation therapy for prostate cancer remains an ongoing clinical challenge and critical quality of life issue. SBRT could minimize rectal toxicity by reducing the volume of rectum receiving high radiation doses and offers the potential radiobiologic benefits of hypofractionation. This study sought to evaluate the incidence and severity of proctitis following SBRT for prostate cancer.

**Methods:**

Between February 2008 and July 2011, 269 men with clinically localized prostate cancer were treated definitively with SBRT monotherapy at Georgetown University Hospital. All patients were treated to 35-36.25Gy in 5 fractions delivered with the CyberKnife Radiosurgical System (Accuray). Rectal bleeding was recorded and scored using the CTCAE v.4. Telangiectasias were graded using the Vienna Rectoscopy Score (VRS). Proctitis was assessed via the Bowel domain of the Expanded Prostate Index Composite (EPIC)-26 at baseline and at 1, 3, 6, 9, 12, 18 and 24 months post-SBRT.

**Results:**

The median age was 69 years with a median prostate volume of 39 cc. The median follow-up was 3.9 years with a minimum follow-up of two years. The 2-year actuarial incidence of late rectal bleeding ≥ grade 2 was 1.5%. Endoscopy revealed VRS Grade 2 rectal telangiectasias in 11% of patients. All proctitis symptoms increased at one month post-SBRT but returned to near-baseline with longer follow-up. The most bothersome symptoms were bowel urgency and frequency. At one month post-SBRT, 11.2% and 8.5% of patients reported a moderate to big problem with bowel urgency and frequency, respectively. The EPIC bowel summary scores declined transiently at 1 month and experienced a second, more protracted decline between 6 months and 18 months before returning to near-baseline at two years post-SBRT. Prior to treatment, 4.1% of men felt their bowel function was a moderate to big problem which increased to 11.5% one month post-SBRT but returned to near-baseline at two years post-SBRT.

**Conclusions:**

In this single institution cohort, the rate and severity of proctitis observed following SBRT is low. QOL decreased on follow-up; however, our results compare favorably to those reported for patients treated with alternative radiation modalities. Future prospective randomized studies are needed to confirm these observations.

## Background

Post-treatment bowel function is a primary determinant of quality of life following radiation therapy for prostate cancer [1-5]. Late proctitis occurs at a frequency of 5–20% with conventionally fractionated treatment [6]. The incidence of proctitis is dependent on the assessment method [7] and whether it is patient or physician reported [8]. Patients with radiation-induced proctitis report increased bowel frequency/urgency, incontinence, bleeding and pain [6]. These symptoms occur months to years after treatment with the large majority of patients reporting symptoms within two years following radiation therapy [9]. Patient characteristics such as age [10], comorbidities [10], hemorrhoids, inflammatory bowel disease [11] and/or anticoagulation [12] may increase an individual’s risk of radiation-induced proctitis. Unfortunately, treatment options for radiation proctitis are limited and of unclear clinical benefit [13].

Proctitis is the principle dose-limiting toxicity of prostate radiotherapy [14-16]. Acute proctitis has been attributed to radiation-induced injury to the epithelial lining of the rectal mucosa, leading to mucosal sloughing, acute inflammatory infiltrates, and vascular permeability leading to edema. These early changes are associated with bowel frequency, bowel urgency and rectal pain. Chronic proctitis results from epithelial atrophy and obliterative endarteritis, resulting in tissue ischemia, rectal fibrosis, stricture formation, and neovascularization; this presents clinically with bowel frequency, urgency and rectal bleeding. Historically, endoscopic findings in patients with proctitis include telangiectasia, congested mucosa, and ulcers. Telangiectasias are observed in 20-80% of patients receiving conventionally fractionated radiation therapy [17].

The risk of proctitis appears to be dependent upon both the total radiation dose and the volume of the rectum in the high dose area [18]. Technical factors such as treatment of the proximal seminal vesicles [19] and expansion of planning target volume (PTV) to compensate for intra-fraction prostate motion [20] may contribute to the severity of rectal toxicities. To minimize bowel toxicity, it is currently recommended that the volume of the rectum receiving > 75 Gray (Gy) be limited to < 10% with conventionally fractionated radiation therapy [21,22]. Studies have shown that advanced radiation technologies such as intensity modulated radiation therapy (IMRT) may decrease the dose to the rectum and potentially decrease proctitis [23,24].

Recent data suggest that large radiation fraction sizes are radiobiologically favorable over lower fraction sizes in prostate cancer [25]. The α/β for prostate cancer may be as low as 1.5 Gy [26]. If the α/β for prostate cancer is less than 3-5 Gy, which is generally the value accepted for late rectal complications, the linear-quadratic model predicts that delivering large radiation fraction sizes will result in improved local control with a similar rate of bowel complications. Therefore, using large fraction sizes in SBRT provides an attractive modality to leverage the potential radiobiologic benefits of hypofractionation with the minimal invasiveness of an external-beam treatment modality [27]. Furthermore, by reducing the number of treatment visits, hypofractionated therapy can also be logistically favorable for patients and reduce financial burden, ultimately decreasing barriers to care.

The safety and efficacy of SBRT in the treatment of clinically localized prostate cancer has been established in a number of studies [28-33]. The use of large fraction sizes in SBRT offers the potential radiobiological benefits of hypofractionation and potentially may minimize radiation proctitis by reducing the volume of the rectum receiving high radiation doses [34]. The CyberKnife robotic radiosurgical system uses image guidance to track implanted fiducials to account for intrafraction prostatic motion [35,36]. This reduces the uncertainty of the location of the prostate and allows treatment to be delivered with a smaller PTV expansion, which may reduce the doses delivered to the anterior rectal wall. The goal of this study is to report the bowel outcomes following SBRT for clinically localized prostate cancer.

## Methods

### Patient selection

Patients eligible for study inclusion had histologically-confirmed prostate cancer treated per our institutional protocol. Clinical stage was defined according to the 6^th^ edition of the American Joint Committee on Cancer criteria. Risk groups were defined using the D’Amico criteria [37]. Internal Review Board (IRB)-approval was obtained for retrospective review of data that was prospectively collected in our institutional database.

### SBRT treatment planning and delivery

SBRT was delivered using the CyberKnife robotic radiosurgical system (Accuray Inc., Sunnyvale, CA). The fiducial placement and computed tomography (CT)/magnetic resonance (MR) simulation procedures have been previously described in Lei *et al*. [36]. The clinical target volume (CTV) was defined as the prostatic capsule and proximal seminal vesicles (to the point where the seminal vesicles separate). To cover areas of potential ECE, the expansion from the CTV to the PTV was 5 mm in all directions except 3 mm posteriorly into the rectum. There was no difference in the target volume delineation for different risk groups. Fiducial-based tracking was used to account for interfraction and intrafraction prostate motion [35]. Treatment planning was performed using Multiplan (Accuray Inc., Sunnyvale, CA). Patients were treated with 35 or 36.25 Gy of radiotherapy delivered in 5 fractions of 7-7.25 Gy each to the PTV, administered over a timespan of 1-2 weeks. This corresponds to a tumor equivalent dose in 2-Gy fractions (EQD2) of approximately 85-90 Gy assuming an α/β ratio of 1.5. The rectum was contoured as a solid structure from the anus (at the level of the ischial tuberosities) to the rectosigmoid flexure and evaluated with dose-volume histogram analysis during treatment planning using Multiplan (Accuray Inc., Sunnyvale, CA) inverse treatment planning. Rectal volume receiving 36 Gy was limited to ≤ 1 cc. Assuming an α/β of 3 Gy for late bowel complications, this is biologically equivalent to approximately 74 Gy administered in 2 Gy fractions. The rectal dose-volume histogram (DVH) goals were <50% rectal volume receiving 50% of the prescribed dose, <20% receiving 80% of the dose, <10% receiving 90% of the dose, and < 5% receiving 100% of the dose. Typical dose distributions have been previously described [28,34,38]. Patients were placed on a low-residual diet and given enemas prior to simulation and treatment delivery to maximize the potential distance between the prostate and the rectal wall and minimize intrafraction prostate motion.

### Follow-up and statistical analysis

Rectal bleeding was prospectively documented at follow-up visits using the National Cancer Institute (NCI) Common Toxicity Criteria (CTC) version 4.0. Acute rectal bleeding was defined as experiencing toxicity within 6 months of radiation therapy. Late rectal bleeding was defined as occurring at least 6 months after delivery of radiation therapy. Grade 1 represents minimal bleeding not requiring medications for symptom control. Grade 2 indicates rectal bleeding requiring new medication (i.e. steroid suppository) or minor laser coagulation. Telangiectasia were graded using the Vienna Rectoscopy Score (VRS): Grade 1 (a single telangiectasia), Grade 2 (multiple non-confluent telangiectasia) and Grade 3 (multiple confluent telangiectasia) [39].

Proctitis was assessed via the bowel domain of the Expanded Prostate Index Composite (EPIC)-26 at baseline and at 1, 3, 6, 9, 12, 18 and 24 months [40]. The timing of the quality of life (QOL) assessments was relative to the last day of SBRT treatment. The EPIC-26 bowel domain includes five questions related to individual symptoms (questions 6a-e: urgency, frequency, pain, bloody stool, incontinence) and one question (question 7) related to overall bother (degree of interference or annoyance caused by bowel symptoms [41].

Wilcoxon signed-rank test and chi-square analysis were used to assess differences in ongoing quality of life scores in comparison to baseline. For each EPIC question, the responses were grouped into three clinically relevant categories (no problem, very small-small problem and moderate to big problem). To statistically compare changes between time points, the levels of responses were assigned a score and the significance of the mean changes in the scores was assessed by Wilcoxon rank test. EPIC scores for the bowel domain and its individual questions range from 0 - 100 with lower values representing worsening bowel symptoms. The minimally important difference (MID) in EPIC score was defined as a change of one-half standard deviation (SD) from the baseline [42]. To limit the effect of attrition bias, statistical analysis was limited to time points in which ≥ 80% of the patient data were available.

## Results

From February 2008 to July 2011, 269 prostate cancer patients were treated per our institutional SBRT monotherapy protocol (Table 1). The median follow-up was 3.9 years. The median age was 69 years and 44.2% were of non-Caucasian ancestry. Comorbidities were common with 35% were taking anticoagulants (including aspirin) prior to treatment. The median prostate volume was 39 cc. By D’Amico classification, 99 patients were low-, 143 intermediate-, and 27 high-risk. 16.4% also received androgen deprivation therapy (ADT) at the discretion of the treating Urologist. 83.3% of the patients were treated with 36.25 Gy in five 7.25 Gy fractions.

**Table 1:** **Baseline patient characteristics and treatment**

**% Patients (N = 269)**		
**Age (y/o)**	Median 69 (44-90)	
	<60	8.20%
	60-69	42.40%
	70-79	41.30%
	≥80	8.20%
**Race**		
	White	55.80%
	Black	37.20%
	Other	7.10%
**Prostate volume (cc)**	Median 39.04	
	(11.56-138.69)	
**Charlson comorbidity index (CCI)**		
	0	66.9%
	1	22.7%
	2	6.7%
	≥3	3.7%
**Body mass index**		
	BMI <25	21.9%
	25 ≤ BMI < 30	47.2%
	30 ≤ BMI < 35	21.9%
	BMI ≥35	8.9%
**Use of anticoagulants**	Yes	35.3%
	No	64.7%
**Risk group**		
	Low	36.80%
	Intermediate	53.20%
	High	10.00%
**Hormonal therapy**		
	Yes	16.40%
	No	83.60%
**Dose**		
	36.25 Gy	83.30%
	35 Gy	16.40%
	Other	0.40%

A total of 61 (22.7%) patients reported rectal bleeding after SBRT, with 28 (10.4%) patients reporting acute bleeding and 38 patients (14.1%) reporting late bleeding. Overall, Grade 2 acute and late rectal bleeding were observed in 0 (0%) and 4 (1.5%) of patients, respectively. There was no Grade 3 or higher rectal bleeds. The majority of acute rectal bleeding occurred within 1-month post-SBRT treatment (79%). The bleeding completely resolved in the majority of the patients by the subsequent follow-up visit. Likewise, the majority of late rectal bleeding was observed at one specific follow-up appointment and did not persist on subsequent follow-ups.

Seventy-three patients had one or more rectal endoscopies during the follow-up period. Endoscopy was performed as a part of normal colorectal cancer screening or if patient had clinically significant rectal bleeding. The median interval from completion of SBRT to endoscopy was 24 months (3 months to 53 months). On endoscopy, rectal telangiectasias were found in 8 (11.0%) patients. All 8 patients were observed to have multiple non-confluent telangiectasias (VRS Grade 2) [38]. No patient had confluent or circumferential telangiectasia. Minor laser coagulation was performed in three patients. No rectal ulcerations, strictures, or fistulas were observed.

**Figure 1:**
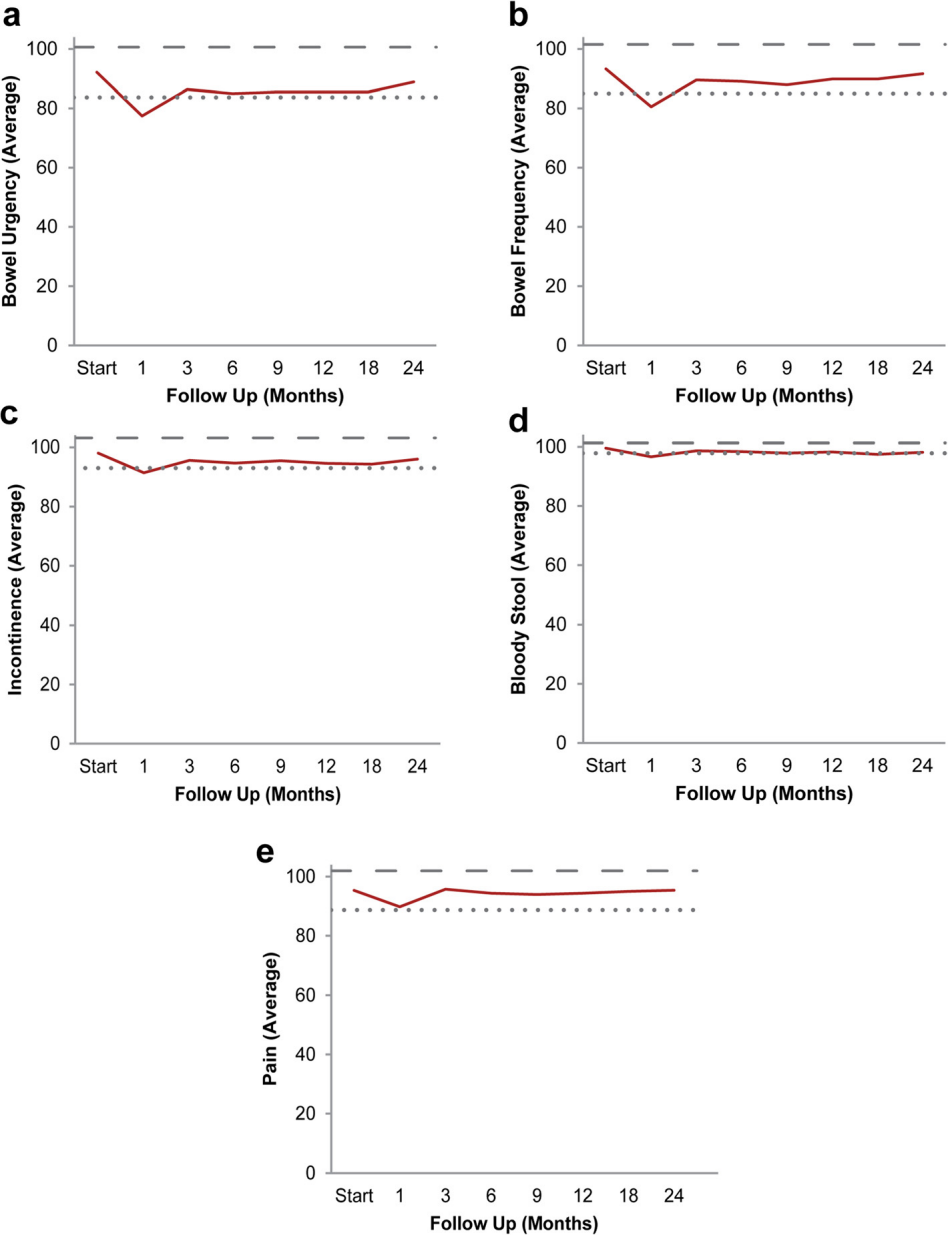
**Individual EPIC-26 bowel symptoms (Questions 6a-e).** Average individual symptom EPIC bother scores at baseline and following SBRT for prostate cancer: **(a)** urgency to have a bowel movement-Question 6a of the EPIC-26; **(b)** increase frequency of bowel movements- Question 6b of the EPIC-26; **(c)** losing control of stools- Question 6c of the EPIC-26; **(d)** bloody stools- Question 6d of the EPIC-26; **(e)** abdominal/pelvic/rectal pain- Question 6e of the EPIC-26. Thresholds for clinically significant changes in scores (½ standard deviation above and below the baseline) are marked with dashed lines. EPIC scores range from 0–100 with higher values representing a more favorable health-related QOL.

At baseline, 1.9% of our cohort reported some level of annoyance due to bloody stools, however no patient felt it was a moderate to big problem (Table 2). The mean changes in EPIC bloody stool bother scores from baseline to 2 years of follow-up are shown in Table 3. The mean EPIC bloody stool bother score was 99.5 at baseline (Table 3). Bloody stool bother increased following treatment with the mean score decreasing to 96.6 at 1 month post-treatment (mean change, -2.9) (*p* = 0.0002) (Table 3, Figure 1d). However, only 1.2% of patients felt that that bloody stools were a moderate to big problem at 1 month following treatment (Table 2). Although bloody stool bother declined quickly, a second late increase in bloody stool bother was observed with the mean bloody stool bother score decreasing to 97.4 at 18 months (mean change from baseline, -2.1) (p = 0.0066) (Table 3, Figure 1d). Both declines met the threshold for clinically significant change (MID = 1.7). However, only 1.2% of patients felt that that bloody stools were a moderate to big problem at 12 months following treatment (Table 2). By two years following SBRT, bloody stool bother returned to near-baseline with bloody stool bother score of 98.1 (mean change from baseline, -1.4). By two years post-SBRT, no patient felt that blood stools was a moderate to big problem (Table 2).

**Table 2:** **Bowel symptoms following SBRT for prostate cancer**

		**Start**	**1**	**3**	**6**	**9**	**12**	**18**	**24**
**Bowel urgency**									
No problem		77.2%	49.8%	62.0%	60.2%	64.5%	62.3%	64.7%	66.8%
Very small-small problem		21.0%	39.0%	33.7%	34.1%	29.8%	31.6%	29.5%	31.1%
Moderate-big problem		1.9%	11.2%	4.3%	5.6%	5.6%	6.1%	5.8%	2.1%
	*p* value		<0.0001	0.0002	<0.0001	0.0002	0.0001	0.0004	0.01568
**Bowel frequency**									
No problem		82.0%	57.0%	71.9%	71.5%	70.6%	72.5%	76.3%	77.4%
Very small-small problem		15.7%	34.5%	25.0%	25.3%	23.4%	23.0%	18.8%	19.7%
Moderate-big problem		2.2%	8.5%	3.1%	3.2%	6.0%	4.5%	4.9%	2.9%
	*p* value		<0.0001	0.0079	0.0038	0.0031	0.0214	0.0169	0.2508
**Incontinence**									
No problem		94.8%	79.5%	85.2%	86.7%	88.3%	85.2%	85.7%	89.5%
Very small-small problem		4.1%	17.0%	14.5%	11.2%	9.7%	12.3%	11.6%	8.8%
Moderate-big problem		1.1%	3.5%	0.4%	2.0%	2.0%	2.5%	2.7%	1.7%
	*p* value		<0.0001	0.0033	0.0021	0.03	0.0001	0.0001	0.0147
**Bloody stools**									
No problem		98.1%	90.8%	96.9%	95.6%	96.0%	96.3%	94.2%	94.2%
Very small-small problem		1.9%	8.1%	2.3%	3.6%	2.4%	2.5%	5.4%	5.8%
Moderate-big problem		0.0%	1.2%	0.8%	0.8%	1.6%	1.2%	0.4%	0.0%
	*p* value		0.0002	0.1677	0.0934	0.0574	0.0906	0.0066	0.0258
**Pain (abdominal, pelvic or rectal)**									
No problem		86.1%	76.8%	89.8%	86.7%	86.7%	88.5%	86.6%	90.3%
Very small-small problem		12.4%	17.8%	7.8%	10.4%	9.7%	8.2%	12.1%	7.1%
Moderate-big problem		1.5%	5.4%	2.3%	2.8%	3.6%	3.3%	1.3%	2.5%
	*p* value		0.0002	0.5516	0.6606	0.232	0.5713	0.8565	0.7498
	***N=***	*267*	*259*	*255*	*249*	*248*	*244*	*224*	*238*

Patient-reported responses to EPIC-26 questions 6A (Urgency to have a bowel movement), 6B (Frequency of bowel movements), 6C (Losing control of your stools), 6D (Bloody stools) and 6E (Abdominal, pelvic or rectal pain).

**Table 3:** **Changes in EPIC bowel summary and overall bowel bother scores following SBRT for prostate cancer**

**EPIC criteria**	**Baseline**	**1 Month**		**6 Month**		**12 Month**		**18 Month**		**24 Month**	
		**Change**	**S.D.**	**Change**	**S.D.**	**Change**	**S.D.**	**Change**	**S.D.**	**Change**	**S.D.**
**Bloody stool bother**	99.5	−2.9	12.26	−1.1	8.54	−1.3	9.86	−2.1	10.93	−1.4	8.03
**Bowel summary**	94.8	−9.8	17.89	−3.2	12.21	−3.5	13.04	−3.3	13.40	−1.6	10.73
**Bowel bother**	90.7	−14.8	26.98	−2.6	20.82	−5.5	23.95	−3.5	20.91	−0.9	18.86

Baseline proctitis symptoms were uncommon in our patients (Table 2). All proctitis symptoms increased at one month post-SBRT. The most bothersome symptoms were bowel urgency and frequency. At one month post SBRT, 11.2% and 8.5% of patients reported moderate to big problems with bowel urgency and frequency, respectively (Table 2). 9.7% of patients required anti-diarrheals within the first month post-SBRT.

**Figure 2:**
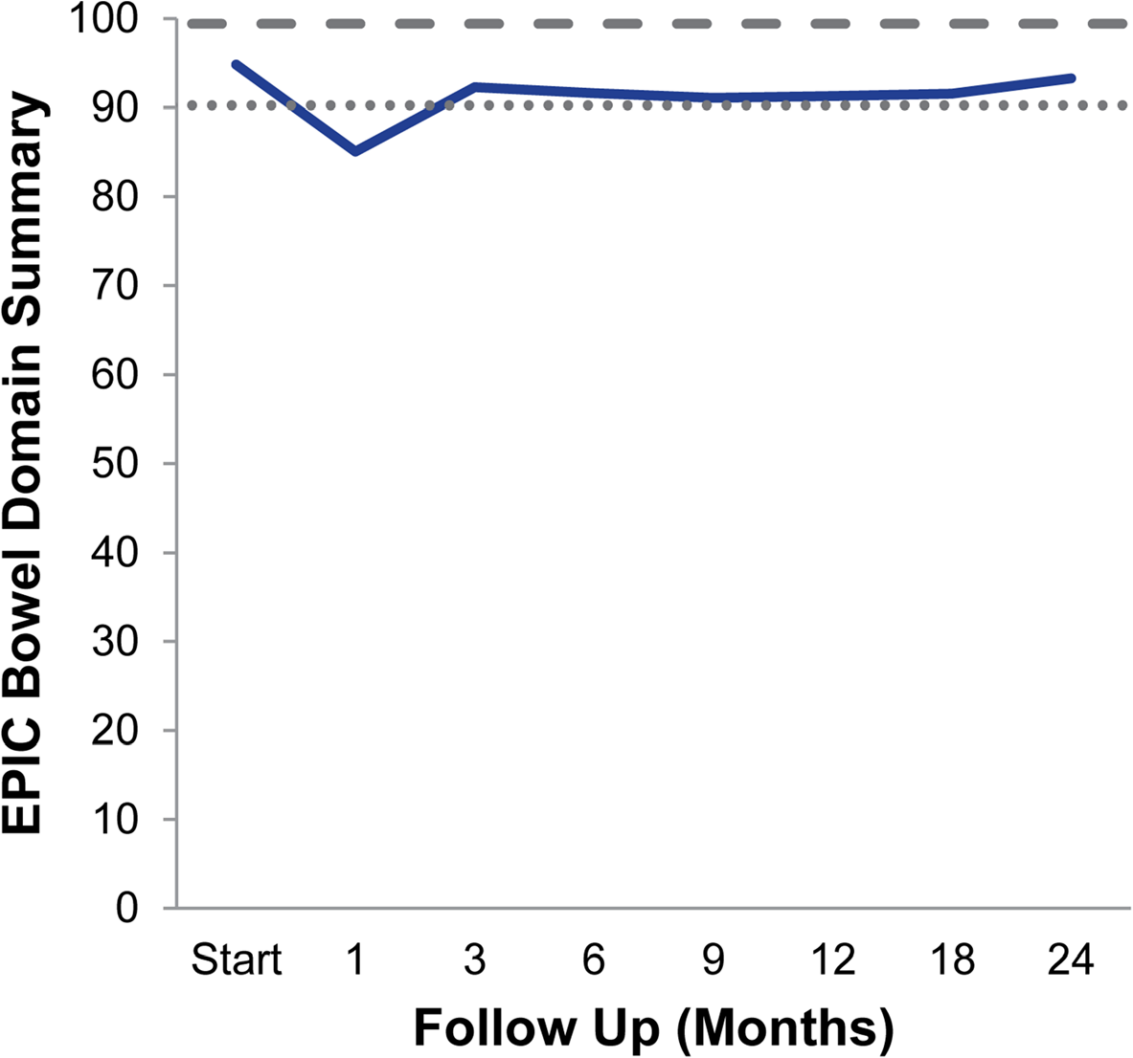
**Average EPIC bowel summary scores at baseline and following SBRT for prostate cancer.** Thresholds for clinically significant changes in scores (½ standard deviation above and below the baseline) are marked with dashed lines. EPIC scores range from 0–100 with higher values representing a more favorable health-related QOL.

Likewise, the EPIC bowel summary score declined transiently at 1 month (mean change, -9.8) (Table 3). In addition, a second more protracted decline occurred between 6 and 18 months (mean change from baseline at 12 months, -3.5). Bowel declines at 1 month and 12 months were statistically significant (*p* < 0.0001); however, only the 1 month change met the threshold for clinically significant change (MID = 4.6) (Figure 2). The EPIC bowel summary score returned to near-baseline at two years post-SBRT (mean change from baseline, -1.6). Individual bowel symptoms changed with time in a similar manner (Figure 1).

**Figure 3:**
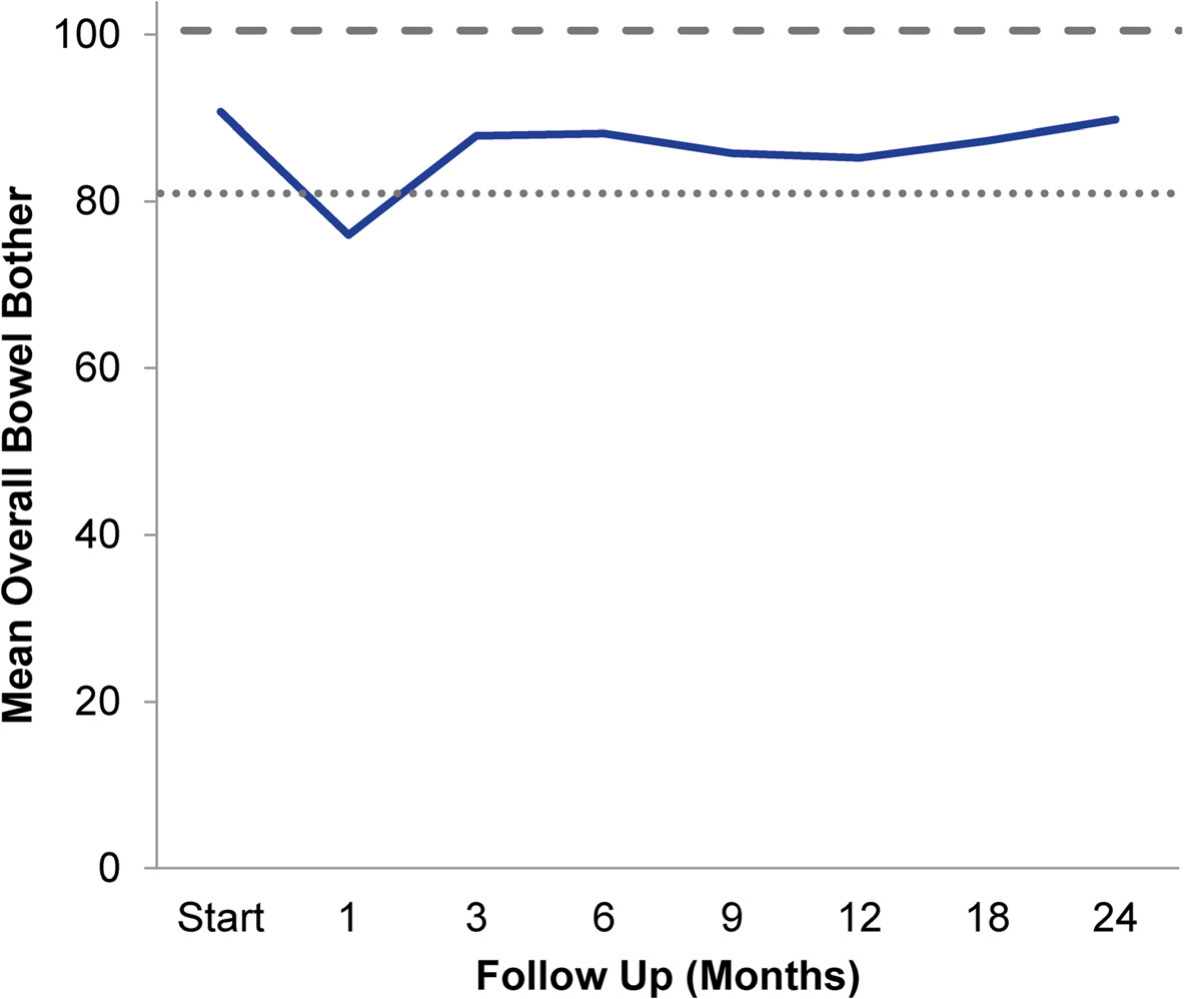
**Overall bowel bother score (baseline and following SBRT; Question 7 of EPIC-26).** Thresholds for clinically significant changes in scores (½ standard deviation above and below the baseline) are marked with dashed lines. EPIC scores range from 0–100 with higher values representing a more favorable health-related QOL.

Overall bowel bother showed a similar pattern as the bowel summary score. At baseline, 24.0% of patients reported some level of annoyance due to bowel symptoms with 4.1% of patients feeling that bowel function was a moderate to big problem (Table 4). The mean EPIC bowel bother score was 90.7 at baseline (Table 3). Overall bowel bother worsened post-treatment and the mean score decreased to 75 at 1 month (mean change, -14.8) (Table 3, Figure 3). However, only 11.5% of patients felt that their bowel function was a moderate to big problem at 1 month following SBRT (Table 4). Bowel bother scores worsened over a second, more protracted time period (Figure 3). Once again, only the first decline met the threshold for clinically significant change (MID = 9.8). Only 7% of patients felt that bowel symptoms were a moderate to big problem at 12 month following treatment (Table 4). By two years following SBRT, overall bowel bother returned to near baseline with a bowel bother score of 89.8 (mean change from baseline, -0.9) (Table 3) and 3.3% of patients feeling bowel symptoms were a moderate to big problem (Table 4).

**Table 4:** **Overall bowel bother following SBRT for prostate cancer (Patient-reported responses to Question 7 of the EPIC-26)**

		**Start**	**1**	**3**	**6**	**9**	**12**	**18**	**24**
No problem		76.0%	44.6%	68.0%	69.1%	63.7%	63.5%	66.1%	70.4%
Very small-small problem		19.9%	43.8%	28.9%	26.9%	30.2%	29.5%	29.5%	26.3%
Moderate-big problem		4.1%	11.5%	3.1%	4.0%	6.0%	7.0%	4.5%	3.3%
	*p* value	-	<0.0001	0.1044	0.0828	0.0051	0.0005	0.0172	0.3499
	***N=***	*267*	*260*	*256*	*249*	*248*	*244*	*224*	*240*

## Discussion

Proctitis following prostate cancer radiotherapy is a critical quality of life issue [1-4] and the principle dose-limiting toxicity [14-16]. Currently, there is limited data on proctitis following SBRT for prostate cancer. A better understanding of bowel symptoms following SBRT would enable clinicians to provide more realistic expectations to patients as they weigh their treatment options. Our prior paper reported the prevalence, severity and overall incidence of bowel frequency/urgency, rectal pain and rectal bleeding following SBRT [28]. However, it is reliant on physician reporting which may under report the incidence of bowel symptoms [8] and provides no information on the associated bother [43,44]. In this study, we utilized the EPIC-26 bowel domain to evaluate bowel symptoms but also assess related bother.

Stereotactic body radiation therapy (SBRT) offers to minimize radiation-associated rectal toxicity by reducing the volume of rectum receiving high radiation doses. The overall incidence of post-SBRT rectal bleeding in this series was 23%. The low rate of late Grade ≥ 2 rectal bleeding (1.5%) seen in this study is consistent with the results from a prior review by our institution [28] and results from other institutions [45,32] which all report a rate of late Grade ≥ 2 rectal bleeding of < 5%. In fact, many of the bleeds were acute and transient suggestive of acute anal irritation or hemorrhoid exacerbation due to bowel frequency [38]. Furthermore, the incidence of telangiectasia in patients who completed post-SBRT rectal endoscopy for the assessment of rectal bleeding or cancer screening was 11% which is significantly lower than the rates reported in prospective studies that looked at endoscopic outcomes after conventionally fractionated radiation therapy [17,46,47].

These findings are particularly significant given that rectal bleeding is one of the principle dose-limiting toxicities, and is thus a potential barrier to administering radiation at appropriately therapeutic levels. In the work by Sanda et al, the proportion of patients reporting rectal bleeding as a moderate-big problem on the EPIC 26 survey progressively increased over time (reaching 5% at 12 and 24 months), ultimately manifesting as a late complication post-treatment. However, we observed that with SBRT rectal bleeding occurred at lower rates and presented as a relatively earlier complication: the incidence of patients reporting rectal bleeding as a moderate-big problem in this study reached a peak at 9 months at 1.6%, and by 24 months the incidence dropped to null. Additionally, in our series the rate of Grade 2-3 late rectal bleeding was 1.5%. This compares favorably to the 5%-15% seen with contemporary high-dose IMRT [24,48]. We speculate that this reassuring profile with SBRT may be a result of the enhanced accuracy with fiducial tracking and narrowed target volumes, thus enhancing our efforts to spare normal tissue from inadvertent irradiation.

**Figure 4:**
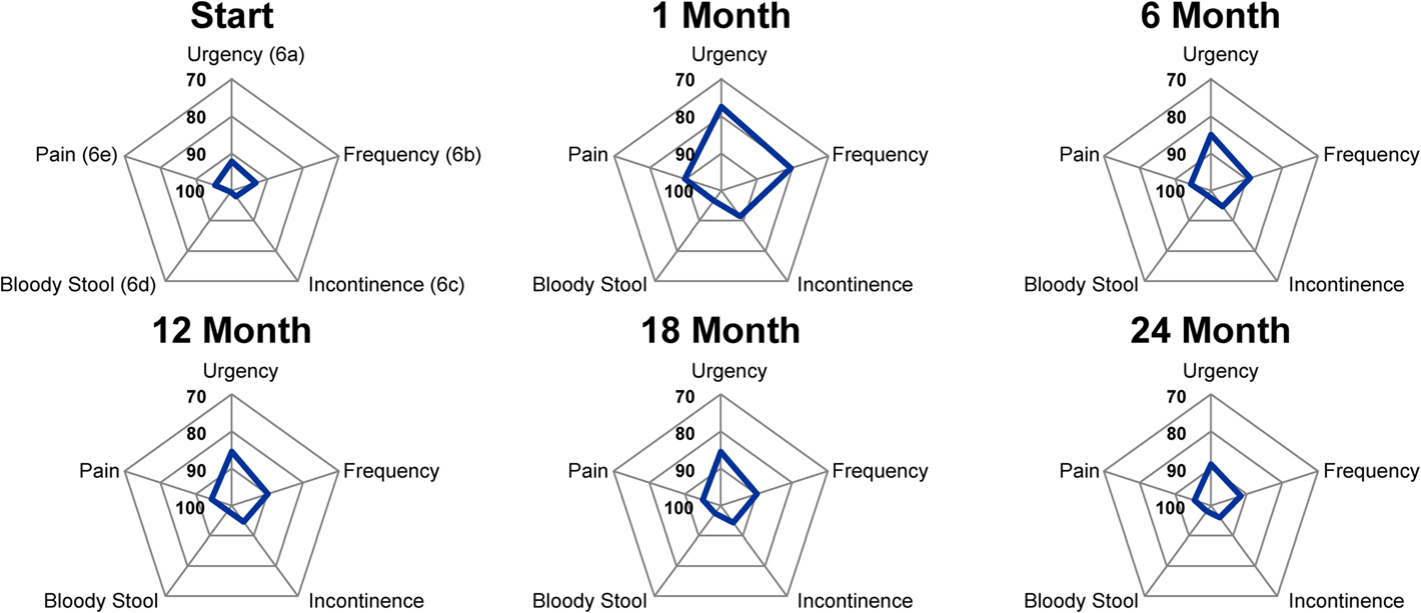
**Radar plots showing the distribution of individual symptom bother following SBRT for prostate cancer.** EPIC scores range from 0–100 with higher values representing a more favorable health-related QOL. Points further out from the center indicate higher levels of bother with a given symptom.

The pattern seen in bowel QOL after SBRT in our study is similar to the pattern seen after conventionally fractionated radiotherapy, proton therapy or brachytherapy. The bowel QOL score is at its lowest 1 or 2 months after treatment, but improves slowly thereafter to near baseline by 1-2 years after treatment [3-5]. In our series, most moderate to big problems were seen at 1 month post-SBRT with approximately 10% of patients reporting moderate to big problems with bowel urgency and/or frequency (Figure 4). This compares favorably with 15-19% incidence of moderate to severe bowel urgency and frequency seen two months after conventionally fractionated external beam radiation therapy or brachytherapy [3]. Unlike conventional modalities, this increase in bowel bother was transient and returned to near baseline by 3 months post-SBRT [3,5]. A second transient increase in bowel bother occurred at 12 months with approximately 7% of patients reporting moderate to big problems. Unlike the pattern seen with conventional modalities, post-SBRT bowel bother returned to near baseline by two years [3,5]. The mean bowel summary score change from baseline to 24 months in this study was -1.6. This change compares favorably to that seen at 24 months with conventionally fractionated IMRT and proton therapy -7.4 and -3.7, respectively [3-5,14].

The present study has several identifiable limitations. The patient population was derived from a single high-volume institution cohort that can limit the translation of our work to the general population. Nevertheless, our work utilized a fairly large patient population with excellent follow-up rates, and was heterogeneous with respect to ethnicity and risk stratification, and considered key patient characteristics such as comorbidity, body mass index, and use of anticoagulants and hormonal therapy. It is conceivable, however, that institutions without the same experience may have a learning curve before they can achieve similar results. Also noteworthy is that our analysis was conducted without a concurrent comparator arm and thus must be carefully weighed against previously published work. Reassuringly, given that the observed toxicity rates in the acute phase are within range of previous studies, the superior toxicity profile at two years follow-up with SBRT compared to other modalities offers intriguing insight to guide subsequent trials for a more comprehensive assessments of SBRT-related proctitis in the future.

## Conclusions

In this single institution cohort, the rate and severity of proctitis observed following SBRT is low. QOL tended to improve with longer follow-up and was near baseline at two years post-SBRT. Our results compare favorably to those reported for patients treated with alternative radiation modalities. Future prospective randomized studies are needed to confirm these observations.

### Consent

This study was a retrospective review of prospectively collected data that was approved by the Georgetown University Institutional Review Board.

## Abbreviations

ADT: Androgen deprivation therapy
CT: Computed tomography
CTC: Common toxicity criteria
CTV: Clinical target volume
DVH: Dose-volume histogram
EQD2: Equivalent dose in 2-Gy fractions
EPIC: Expanded prostate index composite
GTV: Gross target volume
Gy: Gray
IMRT: Intensity modulated radiation therapy
IRB: Internal review board
PTV: Planning target volume
QoL: Quality of life
MID: Minimally important difference
MR: Magnetic resonance
NCI: National cancer institute
SD: Standard deviation
SBRT: Stereotactic body radiation therapy
VRS: Vienna rectoscopy score
